# Motor fiber function in spinal muscular atrophy—analysis of conduction velocity distribution

**DOI:** 10.3389/fneur.2023.1305497

**Published:** 2023-12-07

**Authors:** Magdalena Koszewicz, Jakub Ubysz, Edyta Dziadkowiak, Malgorzata Wieczorek, Slawomir Budrewicz

**Affiliations:** ^1^Department of Neurology, Wroclaw Medical University, Wroclaw, Poland; ^2^Faculty of Earth Sciences and Environmental Management, University of Wroclaw, Wroclaw, Poland

**Keywords:** spinal muscular atrophy, peripheral nerves, polyneuropathy, collision test, conduction velocity distribution

## Abstract

**Objectives:**

The motor neuron survival protein, which is deficient in spinal muscular atrophy (SMA), performs numerous cellular functions. Currently, SMA is believed to be a multi-organ disease, including lesion of various structures of the central and peripheral nervous systems. Motor nerve damage, especially in milder SMA types, is controversial. This prompted the conduct of the electrophysiological studies in adults with SMA types 2 and 3 presented in this paper.

**Methods:**

The study group consisted of 44 adult patients with SMA types 2 and 3. All patients underwent neurological examination with Hammersmith Functional Motor Scale-Expanded (HFMSE) assessment. Standard electrophysiological studies in the ulnar nerve and conduction velocity distribution (CVD) tests were performed in all patients and controls.

**Results:**

A prolongation of the distal latency and lowering of the motor potential amplitude with no changes in CVD were found in the whole patient group. There were no dependencies on the number of gene copies. Patients with low HFSME value had slower standard conduction velocity, CVD in upper and median quartiles, and narrower CVD spread; in milder SMA, CVD spread was greater than in controls.

**Interpretation:**

The significant reduction in motor response amplitude in SMA seems to be primarily related to motor neuron loss and directly proportional to its severity. The coexisting rearrangement in the peripheral nerve structure is present in SMA, and this could be partially caused by a coexisting demyelinating process. Nerve remodeling mainly affects large fibers and occurs in more severe SMA types with significant disability.

## Introduction

Since its first description in the 1890s, spinal muscular atrophy (SMA) has been regarded as a condition in which only alpha motor neurons are damaged, and muscle atrophy and weakness of varying degrees follow (1, 2). The identification of the gene for the most common form of SMA, 5q, and the registration of drugs used for SMA treatment has led to huge interest in the molecular processes responsible for motor neuron damage in this disease entity (1, 3–5). Currently, the *survival motor neuron* (SMN) protein is thought to play various cellular roles and is important in almost all body cells. Patients with SMA are found to have abnormalities in various organs and systems, including other structures of the central and peripheral nervous system (6–8). The activity of alpha motor neurons and, as a result, the contraction of the muscle are subject to regulatory processes and complex feedbacks which are influenced by central nervous system and peripheral sensory inputs (sensory–motor circuit). In SMA, the circuit appears to be defective in its various parts (6, 9, 10). There are experimental studies and case reports indicating damage to sensory nerves and the entire sensory system (6, 9, 11, 12). The assessment of motor nerves and the possible coexistence of motor neuropathy in SMA has been considered by far fewer researchers (11–14). Duman et al. (11) suggested that severe axonal neuropathy could be an additional factor influencing the amplitude of compound motor action potential (CMAP) in patients with SMA type 1. Moosa and Dubowitz (15) described the lowering of motor nerve conduction velocity in the ulnar and tibial nerves in severely affected infants with SMA. In animal models, damage to axons or neuromuscular junctions has been noted as a co-survival or even primary cause of motor deficits (16–18).

The controversy surrounding the coexistence of peripheral motor nerve fiber damage in SMA patients, especially in milder types of the disease, prompted us to conduct electrophysiological studies in adults with SMA types 2 and 3. For this purpose, we used the collision method and the conduction velocity distribution (CVD) test, in addition to standard procedures.

## Methods

We obtained approval for the study from the Ethics Committee of Wroclaw Medical University in Poland. All participants gave their informed consent for participation in the study.

The study group consisted of 44 adult patients (19 female, 25 male) with SMA confirmed by genetic testing with the assessment of the number of gene copies. The patients had SMA types 2 and 3, and we distinguished the subgroups 3a and 3b based on the point of disease onset, i.e., before or after 3 years of age, respectively (5). Some of our patients had comorbidities, including some that could affect the results of electrophysiology tests: three had diabetes mellitus and used oral treatment; three patients had supplemented hypothyroidism; one had a hormonally inactive pituitary microadenoma; and one patient suffered from psoriasis. We did not exclude these patients from the study due to the low severity of the above-mentioned diseases and the fact that the number of analyzed patients was limited, which is related to the rare occurrence of SMA. All of the patients were treatment-naive. The control group consisted of 32 healthy volunteers (19 female, 13 male).

All patients underwent clinical neurological examination together with assessment using the Hammersmith Functional Motor Scale-Expanded (HFMSE) (19). Electro-physiological studies were performed in all patients and controls.

The electrophysiological studies were carried out using Viking Select version 7.1.1c. and a Nicolet Biomedical device with multi-mode program (MMP Plus) software which includes a program for the CVD test (Viasys Healthcare Inc., Conshohocken, Pennsylvania, United States). The room temperature was between 21 and 23°C; skin temperature was at least 32°C.

Standard motor nerve conduction (MNC) studies were performed in the left ulnar nerve. We assessed distal latency (DL) of CMAP in milliseconds—ms, amplitude (A) in millivolts—mV, and conduction velocity (V) in meters per second—m/s (20, 21). Points of stimulation were located at the wrist and below the elbow; the recording electrode was placed over the belly of the abductor digiti minimi muscle. The distance between recording and stimulating electrodes was 5.5 cm. During electrical stimulation (supramaximal), we used the current stimulation option, and the duration of the electrical stimulus was 0.2 ms.

In the CVD test, the stimulation was simultaneous at the same points as above (wrist, and below elbow) for the ulnar nerve. We used supramaximal stimulations with the same duration of the electrical stimulus (0.2 ms). The method was based on the collision phenomenon. The interstimulus interval (ISI) was changed according to the distance between the points of stimulation. ISI was automatically extended by 0.1 ms. The proximal potential gradually increased to the same value as the amplitude of the distal potential. The initially short ISI lengthened gradually, unblocking fibers with smaller diameters and lower conduction velocity. We calculated the lower (10%) and upper (90%) quartiles of conduction velocities, its median (50%) value, and the difference between lower and upper quartiles—the spread of conduction velocities (SCVD). If the amplitude of the motor response in the standard examination was below 1 mV, it was not possible to perform the CVD examination (20, 22–24).

Sensory nerve conduction (SNC) tests were additionally performed in the ulnar nerve according to standard procedures (20, 21), using antidromic technique. Sensory nerve action potentials (SNAP) were obtained from the 5th digit, the electrical stimulation was done at the wrist with maintaining the standard distance between electrodes equal 12 cm. Distal latency (in milliseconds—ms), amplitude (in microvolts—uV), and conduction velocity (in meters per second—m/s) were assessed.

In the statistical analysis, we used STATISTICA 13.0 software. The analysis included the number of cases (N), mean values (X), median (M), and standard deviations (SD) of the continuous parameters. We used the Shapiro–Wilk test for assessment of the normality of distribution. T-Student and Mann–Whitney U tests were applied, depending on the distribution of variables, for comparative analysis of the mean values in the patient and control groups. ANOVA was used to evaluate the variance, and in the absence of a normal distribution in the subgroups, the Kruskall–Wallis test was performed instead of ANOVA. A ratio test was performed to assess whether the control group matched the patient group in terms of gender and age structure. Due to the lack of normality of distribution in this subgroup of some variables, Spearman’s rank correlation coefficient was calculated. All tests were conducted at the significance level of *α* = 0.05, with Bonferroni adjustment.

## Results

The mean age of the 44 (19 female, 25 male) SMA patients was 36.09 ± 10.98 years; this was lower than in the control group (44.31 ± 13.03), which consisted of 19 females and 13 males. Based on the ratio test, both groups were not significantly different in terms of gender (*p* = 0.16). All patients except one and all volunteers were right-handed.

The distinguished SMA types—2, 3a and 3b—were represented by nine (women = 7, men = 2), 21 (women = 7, men = 14) and 14 people (women = 5, men = 9), respectively. Twenty-seven patients had three gene copies, 16 had four and one patient had two copies of the SMN gene. The demographics of the study group are shown in Table 1.

**Table 1:** Demographics of the SMA patient group.

	Type 2 *n* = 9		Type 3a *n* = 21		Type 3b *n* = 14	
	Mean	SD	Mean	SD	Mean	SD
Age (years)	30.11	8.02	35.38	11.75	41.00	9.77
onset of symptoms (years)	1.17	0.50	2.33	0.80	10.79	3.68
HFSME (points)	2.56	2.07	16.00	15.51	33.57	17.43
Gene copies (n)	2 copies- 1 3 copies- 7 4 copies - 1		2 copies- 0 3 copies-12 4 copies- 9		2 copies- 0 3 copies- 8 4 copies- 6	

n, number of subjects; SD, standard deviation.

### Standard MNC, CVD, and SNC in the SMA and control groups

Standard MNC in the left ulnar nerve revealed significant prolongation of the distal latency in the SMA group compared with the controls, and significant differences between the study groups (<0.00001) were found in the amplitude of the motor responses. No differences were found in the motor conduction velocity values in the ulnar nerve on the forearm or in the F wave latency (Table 2). We also performed calculations excluding patients with the comorbidities described above; this did not significantly change the previously obtained results.

**Table 2:** Standard motor conduction study in the patient group with SMA and controls.

Ulnar nerve	SMA *n* = 44		Controls *n* = 32		*P*
	Mean	SD	Mean	SD	
Motor DL (ms)	3.24	1.19	2.57	0.36	0.003
Motor A (mV)	3.68	3.09	8.62	1.91	<0.00001
Motor V (m/s)	60.45	9.44	64.39	7.71	0.169
F wave (ms)	29.45	5.63	27.26	2.40	0.145

n, number of subjects; SD, standard deviation; ms, milliseconds; mV, millivolts; m/s, meters per second; DL, distal latency; A, amplitude; V, conduction velocity.

The assessment of CVD parameters did not reveal any differences between SMA patients and controls in the lower (10%) or upper (90%) quartiles of conduction velocities, median (50%) values, or SCVD (Table 3).

**Table 3:** CVD in the patient group with SMA and controls.

Ulnar nerve	SMA *n* = 44		Controls *n* = 32		*P*
	Mean	SD	Mean	SD	
CVD 10%	52.13	8.15	50.80	5.83	0.444
CVD 50%	57.80	8.91	55.96	5.63	0.318
CVD 90%	61.78	9.46	59.26	5.39	0.188
SCVD (90–10%)	9.14	4.45	8.46	3.57	0.324

n, number of subjects; SD, standard deviation; CVD, conduction velocity distribution; SCVD, spread of conduction velocities.

A standard neurographic examination of sensory fibers was also performed in the SMA patient group and the control group. The obtained results are shown in Table 4. The values of latency and sensory conduction velocity were significantly worse in the study group, while the obtained mean amplitude was significantly higher than in the control group. A detailed analysis of the above results along with quantitative sensory testing (QST) will be discussed in a subsequent article.

**Table 4:** Standard sensory conduction study in the patient group with SMA and controls.

Ulnar nerve	SMA *n* = 44		Controls *n* = 32		*P*
	Mean	SD	Mean	SD	
Sensory L (ms)	2.26	0.37	2.10	0.32	0.019
Sensory A (uV)	67.96	27.23	35.34	17.25	<0.00001
Sensoryr V (m/s)	49.13	7.02	54.66	6.19	0.0007

n, number of subjects; SD, standard deviation; ms, milliseconds; uV, microvolts; m/s, meters per second; L, latency; A, amplitude; V, conduction velocity.

### Standard MNC and CVD in relation to the number of gene copies

None of the above-mentioned parameters of standard neurographic examination and CVD showed differences in relation to the number of gene copies, i.e., 3 and 4. We excluded one patient with two copies (Table 5).

**Table 5:** Comparison of the electrophysiological data in groups of patients with 3 and 4 copies of SMN gene.

Ulnar nerve	3 copies *n* = 27		4 copies *n* = 16		*P*
	Mean	SD	Mean	SD	
Motor DL (ms)	3.41	1.19	2.96	1.22	0.076
Motor A (mV)	3.83	3.06	3.58	3.26	0.860
Motor V (m/s)	60.56	10.44	60.38	8.15	0.953
F wave (ms)	29.76	6.71	29.04	3.83	0.808
CVD 10%	52.91	8.88	51.23	7.29	0.557
CVD 50%	58.53	9.24	56.91	8.90	0.608
CVD 90%	62.54	9.42	60.49	10.09	0.539
SCVD (90–10%)	9.24	4.45	8.51	4.36	0.633

n, number of subjects; SD, standard deviation; ms, milliseconds; mV, millivolts; m/s, meters per second; DL, distal latency; A, amplitude; V, conduction velocity; CVD, conduction velocity distribution; SCVD, spread of conduction velocities.

### Standard MNC and CVD in the relation to the SMA type

The evaluation of data for the individual SMA types did not reveal any differences in age and gene copies. Those patients with more severe types of SMA (types 2 and 3a) had significantly worse results in HFSME.

A significant difference in CMAP amplitude was noted between groups, very low in patients with type 2 (Table 6). A comparative analysis of HFSME score and CMAP amplitude was performed in individual SMA types (2, 3a, and 3b). For the listed variables, there was no difference between subtypes 3a and 3b, but there were significant differences between type 2 and the two other SMA types (3a and 3b; Tables 7, 8). The distal latencies, conduction velocities, and CVD values did not differ significantly between groups (Table 6).

**Table 6:** Comparison of the clinical and electrophysiological data in groups of patients with types 2, 3a and 3b SMA.

	Type 2 *n* = 9		Type 3a *n* = 21		Type 3b *n* = 14			*p*-value*
	Mean	SD	Mean	SD	Mean		SD	
Age (years)	30.11	8.02	35.38	11.75	41.00	9.77		0.0728
Gene copies (n)	3.00	0.50	3.48	0.51	3.36	0.50		0.0959
HFSME (points)	2.56	2.07	16.00	15.51	33.57	17.43		<0.00001
Ulnar motor DL (ms)	3.85	1.62	3.04	0.85	3.15	1.26		0.4717
Ulnar motor A (mV)	0.62	0.35	3.52	2.51	5.87	3.19		<0.00001
Ulnar motor V (m/s)	56.33	8.40	60.05	4911.20	63.71	5.90		0.1591
CVD 10%	52.28	5.06	51.48	9.27	53.08	7.44		0.883
CVD 50%	55.50	4.75	57.00	9.43	59.74	9.20		0.480
CVD 90%	59.60	6.63	60.84	9.65	63.91	10.12		0.883
SCVD (90–10%)	7.35	6.83	8.90	4.15	10.06	4.27		0.480

*Kruskal–Wallis test. n, number of subjects; SD, standard deviation; ms, milliseconds; mV, millivolts; m/s, meters per second; DL, distal latency; A, amplitude; V, conduction velocity; CVD, conduction velocity distribution; SCVD, spread of conduction velocities.

**Table 7:** Comparison of the HFSME variable in SMA subtypes.

HFSME SMA type	Type 2	Type 3a	Type 3b
Type 2	X	0.010	0.000
Type 3a	0.010	X	0.053
Type 3b	0.000	0.053	X

SMA, spinal muscular atrophy; HFSME, Hammersmith Functional Motor Scale-Expanded.

**Table 8:** Comparison of the CMAP amplitude variable in SMA subtypes.

Ulnar motor A (mV) SMA type	Type 2	Type 3a	Type 3b
Type 2	X	0.002	0.000
Type 3a	0.002	X	0.270
Type 3b	0.000	0.270	X

SMA, spinal muscular atrophy; A, amplitude; mV, millivolts; CMAP, compound motor action potential.

In 3a and 3b SMA, the changes in CMAP amplitude positively correlated with HFSME score in both groups (0.633 and 0.549, respectively), and with CVD parameters mainly in 3b group (CVD50%—0.499, CVD90%—0.615, SCVD—0.573).

### Standard MNC and CVD in the relation to HFSME

**Figure 1:**
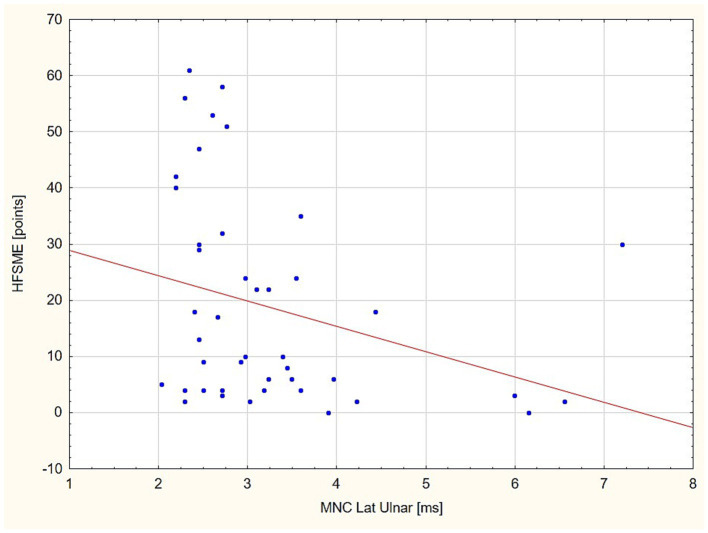
Linear relationship between distal latency (DL) value in the ulnar nerve and HFSME score. HFSME, Hammersmith Functional Motor Scale-Expanded; MNC Lat., distal latency of motor nerve conduction; ms, milliseconds.

**Figure 2:**
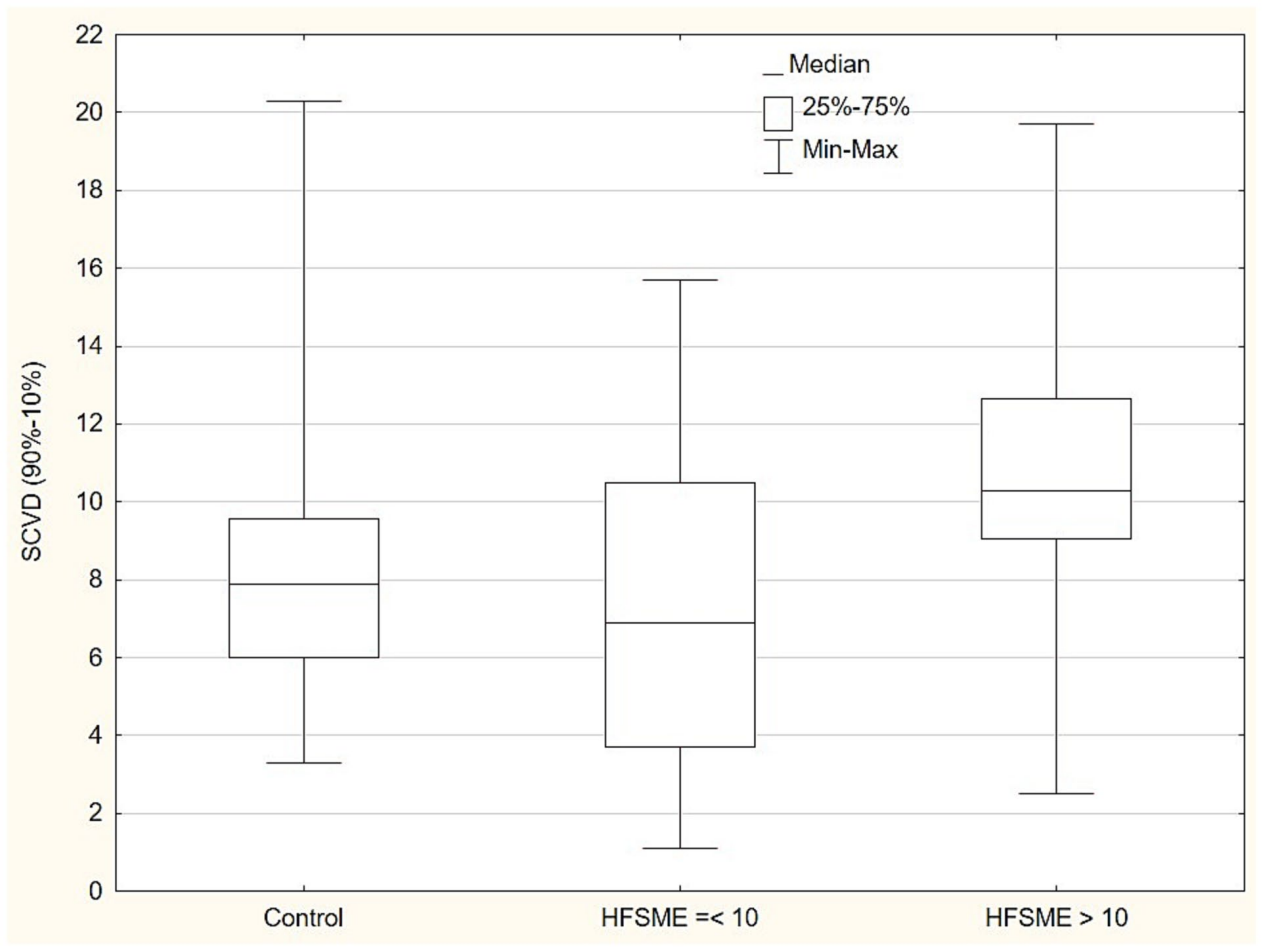
Comparison of conduction velocity spread (SCVD - 90-10% CVD) between SMA patients with HFSME scores above 10 points, below 10 points and controls. SCVD, Conduction velocity spread; HFSME, Hammersmith Functional Motor Scale-Expanded.

In the next step of the statistical analysis, we divided the patient group into two subgroups in the respect to HFSME score: below and above 10 points. The results of the motor conduction velocity (MCV) study were significantly worse (in amplitude and conduction velocity) in patients with low HFSME scores, i.e., below 10 points. Distal latencies in a standard neurographic examination showed a linear relationship with the HFSME value; the higher the score on the scale, the shorter the latencies (Figure 1). Additionally, in patients with low HFSME score CVD50%, CVD90%, and SCVD were lower than in patients with best results in HFSME. Using the Benferroni adjustment, the significance of the differences were kept in CMAP amplitude and SCVD (Table 9). A comparison of SCVD between SMA patients with HFSME scores above 10 points and controls revealed significantly larger spread of conduction velocity (10.63 ± 3.88 v. 8.46 ± 3.57 m/s, *p* = 0.013) in SMA patients (Figure 2).

**Table 9:** Electrophysiological parameters in the SMA patient subgroup with scores below and above 10 points.

Ulnar nerve	HFSME ≤10 points		HFSME >10 points		*P*
	mean	SD	mean	SD	
Motor DL (ms)	3.47	1.23	2.99	1.11	0.061
Motor A (mV)	1.78	1.58	5.75	3.02	0.00002*
Motor V (m/s)	57.00	8.86	64.24	8.75	0.009
F wave (ms)	30.48	5.10	28.58	6.03	0.094
CVD 10%	49.65	9.01	54.24	6.89	0.089
CVD 50%	54.08	8.44	60.96	8.22	0.017
CVD 90%	57.58	8.83	65.36	8.65	0.011
SCVD (90–10%)	7.39	4.54	10.63	3.88	0.025*

*Bonferroni adjustment. HFSME, Hammersmith Functional Motor Scale-Expanded; n, number of subjects; SD, standard deviation; ms, milliseconds; mV, millivolts; m/s, meters per second; DL, distal latency; A, amplitude; V, conduction velocity; CVD, conduction velocity distribution; SCVD, spread of conduction velocities.

## Discussion

The results of the MNC study of the ulnar nerve in patients with SMA types 2 and 3 revealed a significant reduction in the CMAP amplitude compared to controls, similar to the results of other authors (11–13). Significant differences were also shown between patients with different types of SMA, i.e., between types 2, 3a and 3b. The mean value of CMAP amplitude in the patients with SMA type 3b was high, nearly achieving the normal range (5.87 mV), but despite this it was significantly lower than in controls. The pathomechanism of CMAP amplitude reduction in SMA is not clearly explained. Duman et al. (11) emphasized that severe axonal neuropathy might accompany motor neuron loss, and both could account for the markedly reduced CMAP in SMA type 1 patients. We observed a positive correlation between CMAP amplitude reduction and types of SMA and HFSME scores, i.e., less severe symptoms of the disease were connected with higher CMAP amplitudes. On the other hand, a higher CMAP amplitude positively correlated with better CVD values in patients with SMA type 3. The results indicate a very close dependence on the degree of disability and CMAP amplitude, and probably general nerve function. Studies conducted mainly on animal models have suggested that loss of motor neurons follows the primary defect at the axon or the neuromuscular junction (NMJ) in the course of abnormal maturation or disturbances in a feedback loop (7, 8, 13, 17, 25). Ros et al. (26) revealed a decremental response as a sign of postsynaptic dysfunction in 49–60% of SMA patients. Neuropathological findings in patients with SMA type 1 showed delayed maturation of acetylcholine receptor subunits and abnormally formed NMJ. The modern approach tells us to consider SMA as a network disorder. The network consists of central and peripheral parts, but the contribution of dysfunction of the individual portions of the motor circuit to the clinical phenotype remains unknown (16, 26–28).

Evidence of peripheral nerve demyelination in SMA is questionable. Yonekawa et al. (12) related the slowing of conduction velocity in SMA type 1 to the loss of the fastest-conducting fibers rather than to the process of demyelination. Duman et al. (12) indicated that demyelination could be secondary to axonal polyneuropathy or an unknown genetic defect causing both axonal loss and demyelination. In our study, the distal latency in the SMA group was significantly longer than in the control group but did not exceed 50% of the limit of normal values, according to diagnostic criteria for demyelinization by Van den Bergh et al. (29). We found no differences in DL between the groups of patients with different SMA types, different number SMN gene copies, and with low or high HFSME scores. Selective loss of fast-conducting axons could be considered a cause of the slight DL prolongation (30, 31).

In the standard motor conduction study, we did not find a slowing of motor conduction velocity in SMA patients. Additionally, the CVD-based study did not show abnormalities in any of the analyzed compartments, i.e., we did not show any changes to either slow- or fast-conducting motor fibers and SCVD. The patient and control groups were comparable, which was similar to previous published findings (23, 24). We were also unable to find any correlation between these parameters and the number of SMN gene copies or SMA type. Comparing the patient groups with different HFSME values, we revealed significant slowing of the conduction velocity in patients with low scores. In that group, CVD50%, CVD90% and SCVD were significantly slower. The electrophysiological results could indicate a predominant loss of fast-conducting, large fibers in severely ill patients and in consequence with the reorganization of motor nerves. The findings could also provide an explanation for the DL elongation by this phenomenon in the absence of F-wave latency prolongation, which shows a linear, independently proportional dependence on the HFSME score. However, we additionally found an unexpected phenomenon. SCVD values in patients with better HFSME results, i.e., in milder stages of SMA, ranged widely, and significantly wider than in the control group (Figure 2). Given all the previously discussed findings and the suggestion of the dominant loss of fast-conducting fibers, we can explain this finding by the presence of demyelination and an increase in the number of motor fibers with lower conduction velocity values in the early stage of SMA. The presence of a demyelination process of peripheral nerves is also indicated by the results of sensory fiber testing in the form of a significant prolongation of latency and slowing of the sensory conduction velocity. Partially similar findings were discribed by Duman et al. (11) in very young children in age between 1.5 and 26 month.

We assume that, apart from the loss of alpha motor neurons, the rearrangement in the structure of the peripheral nerve in terms of the proportion of motor fibers with different diameters and different rates of motor conduction might take place. Henneman et al. (32) indicated that the diameter of a motor nerve fiber is related to the number of innervated muscular fibers. Other studies have proved that motor neuron size correlates with fiber diameter, number of supplying muscle fibers, and motor unite size (33–35). Many authors underline the size-based vulnerability of motor neurons in amyotrophic lateral sclerosis (ALS) and SMA: motor neurons that are larger and innervate distal muscles are assumed to be more sensitive (33, 36). Our study is able to partially confirm these previous findings, because CVD results revealed a preponderance of damage to larger fibers (CVD90%) in more severe forms of SMA. Slowing of conduction velocity in terms of medium value (CVD 50%) and very wide SCVD in the early stage of SMA supports the idea of the coexistence of motor fiber demyelination in this stage of the disease. Summing up the above observations, it seems that peripheral nerves are damaged in the early stages of the disease, i.e., we can recognized polyneuropathy with demyelination in its image.

The limitations of the study include the small group of patients and the neurophysiological study limited to one nerve. The choice of the nerve was dictated by its relatively best feature in relation to other nerves in the initial screening and the relatively small deformities of the upper limb, which would make it difficult to conduct a collision study. In the study we intended to relate the neurographic findings to the patients’ general condition, hence the choice of the HFSME scale rather than, for example, the Revised Upper Limb Module (RULM). RULM is intended for the assessment only upper limb function. An additional factor was that in the RULM scale, patients relatively easily settle on the so-called “ceiling effect,” which equalizes the scores of individual patients even with significantly different degrees of disability. In addition, the study included patients who had comorbidities that favor the development of polyneuropathy, but this has been explained above. The most important limitation was the differing mean ages of the patient and control groups. However, in both groups, the mean age range was in the fourth-fifth decade of life. In this range, age has no significant effect on the values of electrophysiological parameters. In the literature, a decreasing trend in results from neurophysiological results is clearly observed only in the age group ≥46 years (37).

In conclusion, it appears that the significant reduction in motor response amplitudes in SMA patients seems to be primarily related to motor neuron loss and is directly proportional to its severity. We have been able to provide evidence of coexisting rearrangement in the peripheral nerve structure in terms of the proportion of motor fibers with different diameters and different rates of motor conduction. This process may be partially caused by a coexisting, small demyelinating process already present in the early stage of the disease. Nerve remodeling mainly affects large fibers and occurs in more severe stages of SMA with significant disability.

## Data availability statement

The original contributions presented in the study are included in the article/supplementary material, further inquiries can be directed to the corresponding author.

## Ethics statement

The studies involving humans were approved by the Ethics Committee in Wroclaw Medical University. The studies were conducted in accordance with the local legislation and institutional requirements. The participants provided their written informed consent to participate in this study.

## Author contributions

MK: Conceptualization, Supervision, Writing – original draft. MW: Data curation, Formal analysis, Writing – review & editing. SB: Conceptualization, Funding acquisition, Writing – review & editing. JU: Acquisition of clinical data, Analysis of data. ED: Acquisition of electrophysiological data, Interpretation of data.
